# Supplementary material for: Repurposing niclosamide ethanolamine for alveolar echinococcosis reveals a disconnect between in vitro efficacy and in vivo outcome in the intraperitoneal infection model
Source: PLoS Negl Trop Dis. 2026 Aug 31;20(8):e0014016. doi: 10.1371/journal.pntd.0014016 (PMC13552948; doi:10.1371/journal.pntd.0014016)
Supplement: Supplementary file 1 [file pntd.0014016.s007.docx]

**Comments on Manuscript No. PNTD-D-26-00251**

**Repurposing Niclosamide Ethanolamine for Alveolar Echinococcosis Reveals a Disconnect Between In Vitro Efficacy and In Vivo Outcome**

**Methods**

-Are the objectives of the study clearly articulated with a clear testable hypothesis stated?
-Is the study design appropriate to address the stated objectives?
-Is the population clearly described and appropriate for the hypothesis being tested?
-Is the sample size sufficient to ensure adequate power to address the hypothesis being tested?
-Were correct statistical analysis used to support conclusions?
-Are there concerns about ethical or regulatory requirements being met?

The objective is well articulated; that of repurposing NEN from the field of oncology to the treatment of echinococcosis. This is based on the fact that previous studies have demonstrated high liver concentrations of 1.5 µM within 4 h in mice treated with 40 mg/kg. This is in contrast to niclosamide, an FDA-approved anthelmintic that has shown poor gastrointestinal absorption and bioavailability preventing effective penetration and elimination of tissue-dwelling parasites.

Ethical approval was obtained for the conduct of the study. Requirements were met.

Study design: Evaluation of NEN and other available drugs to determine the In Vitro Efficacy and In Vivo Outcomes was well planned.

The authors to consider presentation of the methodology section under two headings for clarity: In Vitro Efficacy and In Vivo Outcome. This will avoid repetitions and enhance information flow and readership. In some areas, especially grouping of experimental animals, I suggest that tables be used, well labelled. These will also assist in presentation of results/findings for clarity.

All tables: must strictly adhere to PLONTD Journal standards. Each Table should self-explanatory, with clear titles. Any abbreviations used should be explained in the table legend.

Methodology:

1.	Chemicals and Reagents

2.	In vitro Efficacy

Parasite source and maintenance (Lines 146-148)

Line 146: Provide a short description of are the H95 parasites? Indicate source.

In vitro culture of metacestode vesicles (lines 149-155)

Assessment of NEN-induced metacestode vesicle damage (lines 157-158; delete "..by phosphoglucose isomerase

assay"). This is well-captured in the text.

E. multilocularis GL cell isolation (line 176)

- Replace GL with germinal layer in the title)

Methods on in vitro Efficacy to be included in this section are well detailed.

Line 199:

Assay used has no reference

2). At Line 284 insert: In vivo Outcome

- Experimental animals; to include ethical clearance, description, source, and maintenance

Re-organize this section (refer to In Vitro Efficacy section).

**Results**
-Does the analysis presented match the analysis plan?
-Are the results clearly and completely presented?
-Are the figures (Tables, Images) of sufficient quality for clarity?

As presented, the results are detailed. Re-organization of the text will avoid repetition of methodology. This will also assist in Discussion and Conclusion aligned not only to the main Objective of the study but also the Manuscript Title: "Repurposing Niclosamide Ethanolamine for Alveolar Echinococcosis Reveals a Disconnect Between In Vitro Efficacy and In Vivo Outcome".

Suggestion: that the presentation of Results follows the re-organized Methodology section.

1. In vitro Efficacy

- Line 365 information already in methodology. Either delete or re-phrase to read " Following assessment of the efficacy of ...using PGI assay,...."

Line 397: Ensure Table 1 is in strict adherence to the PLOSNTD Journal Quality Standards on presentation of Tables and Figures

2. In vivo Outcome

- Line 431-432 Suggested Title: A pharmacokinetic-pharmacodynamic (PK/PD) mismatch

- Lines 433-535 - Delete. Move to Methodology or re-phrase

3. Comparison of In Vitro Efficacy and In Vivo Outcome

Review the legend information for each figure (e.g Fig. 1) and remove information that is not necessary, for clarity.

E.g.

Legend: Lines 830-843

Fig 1. Niclosamide and NEN damage E. multilocularis metacestode vesicles and reduce the viability of GL cells in vitro.

Suggested Title: Fig. 1. Effect of niclosamide and NEN on E. multilocularis metacestode vesicles in vitro

This legend is too long, needs to be reviewed. Most of the information on methodology is not necessary.

A, PGI release assay was employed after 5 days of metacestode vesicle treatment to assess the efficacy of niclosamide (dark grey) and NEN (grey) - both at concentrations ranging from 10’000 to 13.7 nM. DMSO solvent controls are subtracted, and all data is presented relative to the PGI values obtained in the presence of 0.1% Tx-100. Four independent assays were conducted each with technical triplicates. Mean values and SDs from all experiments performed are shown. B, representative light microscopic images of metacestode vesicles treated with 370 nM niclosamide, NEN, and ethanolamine, DMSO (0.1%) and Tx-100 (0.1%). Scale bar = 2 mm. C, GL cell viability as assessed by CellTiter Glo assay upon treatment of GL cells for 5 days with a concentration range (10’000 to 4.9 nM) of niclosamide (dark grey), NEN (grey) or ethanolamine (light grey). Data is shown relative to values obtained upon treatment with 0.1% DMSO (100% viability), and mean values and SDs of four independent experiments are shown, all conducted in technical quadruplicates. D, Representative photomicrographs of drug-treated and control-treated GL cells are shown. Scale bar = 100 µm.

**Conclusions** *-Are the conclusions supported by the data presented?
-Are the limitations of analysis clearly described?
-Do the authors discuss how these data can be helpful to advance our understanding of the topic under study?
-Is public health relevance addressed?*

Yes, conclusions are supported by the data presented.

As observed and documented in the Discussion: Line 500-503 - "..treatments with both NEN and ESI-09, and several other compounds active against metacestodes, led to the accumulation of extracellular-like vesicles in the matrix of the LL (Zumstein et al., 2025). This response may represent a broader stress reaction of the parasite and warrants further investigation"

Metacestodes actively modulate the host's immune system to survive by triggering an antibody response. This is done by suppressing cell-mediated immunity, absorbing host antibodies on their surface to disguise themselves, and secreting antigens that "trap" the body's antibodies, making it difficult for the immune system to destroy the cysts.

Could these cysts be related to the accumulation of extracellular-like vesicles observed and reported in this study?

Re-formulating of NEN using nanoparticle-based delivery has been suggested as a solution to the following identified challenges that include poor bioavailability, rapid systemic clearance by the liver, and limited tissue penetration. An investigation into the role of cysts will also be important in efforts to re-purpose NEN.

In addition, pharmacokinetic evaluation using the mouse model has its own limitations, which include low total blood volume, making it difficult to collect the multiple time points needed to create a complete plasma concentration-time curve. This may require search for an alternative animal model in order to generate adequate data required for decision making in efficacy evaluation and dose formulation.

**Summary and General Comments**

The problem is well defined with a clear objective of re-purposing NEN, for the treatment of Alveolar Echinococcosis, a Neglected Tropical Disease, that has no effective treatments in humans. NEN has been investigated for the inhibition of growth of tumors by slowing down cancer cell metabolism. In the current study, data has been generated using the methodologies described, however, there is need to reorganize it in terms of presentation and alignment to the main objective and the manuscript title.
