# Supplementary material for: Repurposing niclosamide ethanolamine for alveolar echinococcosis reveals a disconnect between in vitro efficacy and in vivo outcome in the intraperitoneal infection model
Source: PLoS Negl Trop Dis. 2026 Aug 31;20(8):e0014016. doi: 10.1371/journal.pntd.0014016 (PMC13552948; doi:10.1371/journal.pntd.0014016)
Supplement: Supplementary file 2 [file pntd.0014016.s009.docx]

Dear editor, dear reviewers,

We thank the editors and the reviewers for their time and helpful suggestions to improve our manuscript. Below we go through the comments point by point (our comments are in green) and refer to the line numbers in the track-change version of the manuscript.

**Journal Requirements:**

If the reviewer comments include a recommendation to cite specific previously published works, please review and evaluate these publications to determine whether they are relevant and should be cited. There is no requirement to cite these works unless the editor has indicated otherwise.

1) Please ensure that the CRediT author contributions listed for every co-author are completed accurately and in full.
   At this stage, the following Authors/Authors require contributions: Matías Preza, Nicole Dietrich, Pascal Zumstein, Judith Steinmann, Lea Hiller, Trix Zumkehr, Tobias Kämpfer, Marylène Chollet-Krugler, Laura Vetter, Andrew Hemphill, Sarah Dion, and Britta Lundström-Stadelmann. Please ensure that the full contributions of each author are acknowledged in the "Add/Edit/Remove Authors" section of our submission form.
   The list of CRediT author contributions may be found here: <https://journals.plos.org/plosntds/s/authorship#loc-author-contributions>

=> contributions are now added also in the online system. Thank you for the hint!

2) We do not publish any copyright or trademark symbols that usually accompany proprietary names, eg ©,  ®, or TM  (e.g. next to drug or reagent names). Therefore please remove all instances of trademark/copyright symbols throughout the text, including:
   - ® on pages: 12, and 13
   - TM on pages: 10, 13, and 14.

=> this was changed as suggested

3) Please amend your detailed Financial Disclosure statement. This is published with the article. It must therefore be completed in full sentences and contain the exact wording you wish to be published.
i) State what role the funders took in the study. If the funders had no role in your study, please state: "The funders had no role in study design, data collection and analysis, decision to publish, or preparation of the manuscript."
ii) If any authors received a salary from any of your funders, please state which authors and which funders..
If you did not receive any funding for this study, please simply state: u201cThe authors received no specific funding for this work.u201d

=> this is included in the financial statement.

“This study was supported by grants to BLS from the Uniscientia Foundation (salary PZ) and the Gottfried and Julia Bangerter-Rhyner Foundation, as well as the Swiss National Science Foundation (SNSF) grant numbers 320030-227438 (salary MP) and 320030-236056 (salary TK).

The funders had no role in study design, data collection, analysis and interpretation, decision to publish, or preparation of the manuscript.”

4) We have amended your Competing Interest statement to comply with journal style. We kindly ask that you double check the statement and let us know if anything is incorrect.

=> checked and ok.

5) Your current Financial Disclosure states, “This study was supported by grants to BLS from the Uniscientia Foundation and the Gottfried and Julia Bangerter-Rhyner Foundation, as well as the Swiss National Science Foundation (SNSF) grant number 320030-227438 and 320030-236056. The funders had no role in study design, data collection, analysis and interpretation, decision to publish, or preparation of the manuscript.” However, in your funding information on the submission form, "order of grants" mismatches. Please indicate by return email the full and correct funding information for your study and confirm the order in which funding contributions should appear. Please be sure to indicate whether the funders played any role in the study design, data collection and analysis, decision to publish, or preparation of the manuscript.

=> the order of funders was updated.

=> an email was sent to the editorial office to confirm, and that the funders had no role in the study design, data collection, analysis, decision to publish or preparation of the manuscript.

**Reviewers' Comments:**

**Methods**

Reviewer #1: The present study investigating niclosamide and NEN, assessing the effects of these compounds on Echinococcus multilocularis both in vitro and in vivo in a mouse model, can be considered relevant and is of very high quality in terms of methodology and study design.

Reviewer #2: The experimental design is rigorous with clearly articulated. and the data are of good quality.

Reviewer #3: The objective is well articulated; that of repurposing NEN from the field of oncology to the treatment of echinococcosis. This is based on the fact that previous studies have demonstrated high liver concentrations of 1.5 µM within 4 h in mice treated with 40 mg/kg. This is in contrast to niclosamide, an FDA-approved anthelmintic that has shown poor gastrointestinal absorption and and bioavailability preventing effective penetration and elimination of tissue-dwelling parasites.

Ethical approval was obtained for the conduct of the study. Requirements were met.

Study design: Evaluation of NEN and other available drugs to determine the In Vitro Efficacy and In Vivo Outcomes was well planned.

- Thank you for this overall positive feedback.

The authors to consider presentation of the methodology section under two headings for clarity: In Vitro Efficacy and In Vivo Outcome. This will avoid repetitions and enhance information flow and readership. In some areas, especially grouping of experimental animals, I suggest that tables be used. These will also assist in presentation of results/findings for clarity.

- We added four major subchapters for clarity – as suggested by the reviewer.
- We made the treatment groups more clear by including numberings (i) – (iv) in lines 320-321.

  All tables: must strictly adhere to PLONTD Journal standards. Each Table should self explanatory, with clear title. Any abbreviations used should be explained in the table legend.
- We have spelled out all abbreviations in the table legend and made sure to adhere to the journal guidelines.

  Methodology:
  1. Chemicals and Reagents

  2. In vitro Efficacy
  Parasite source and maintenance (Lines 146-148)
  Line 146: Provide a short description of are the H95 parasites? Indicate source.
- As highlighted in line 144, the name of the isolate was H95; the original source is now included with a reference (Jura et al, 1996).

In vitro culture of metacestode vesicles (lines 149-155)

- As suggested, we included the wording “parasite source” in the title of section 2.1 and split the paragraph so that in vitro culture of metacestodes is now a separate section 2.2.

Assessment of NEN-induced metacestode vesicle damage (lines 157-158; delete "..by phosphoglucose isomerase
assay"). This is well-captured in the text.

- This was removed from the title as suggested (now section 2.3).

E. multilocularis GL cell isolation (line 176)
- Replace GL with germinal layer in the title)

- This was done as suggested.

Methods on in vitro Efficacy to be included in this section are well detailed.

2). At Line 284 insert: In vivo Outcome
- Experimental animals; to include ethical clearance, description, source, and maintenance

Re-organize this section (refer to In Vitro Efficacy section).

- Was implemented as suggested, details on mouse strain, housing and parasite maintenance are given in section 2.1 (lanes 144-154), and the description is now shortened in section 3. It would be incorrect to merge sections 2.1 and section 3.1, as one is for parasite strain maintenance, the other one for in vivo testing of drugs, and different animal licences and specifics apply.

**Results**

Reviewer #1: The results are presented in a structured and clear manner, and the conclusions drawn are appropriate and well supported by the data. The data, figures, and tables are adequate and correctly labeled.

Reviewer #2: The results are clearly presented, especially, the “in vitro–in vivo disconnect” is an important cautionary observation in antiparasitic drug development.

Reviewer #3: Suggestion: that the presentation of Results follows the re-organized Methodology section.

As presented, the results are detailed. Re-organization of the text will avoid repetition of methodology. This will also assist in aligning the Discussion and Conclusion not only to the main Objective of the study but also the Manuscript Title: "Repurposing Niclosamide Ethanolamine for Alveolar Echinococcosis Reveals a Disconnect Between In Vitro Efficacy and In Vivo Outcome".

Results:

1. In vitro Efficacy
- Line 365 information already in methodology. Either delete or re-phrase to read " Following assessment of the efficacy of ...using PGI assay,...."

- We prefer to keep the first introductory sentence (lane 376) for clarity and completeness of the section.

Line 397: Ensure Table 1 is in strict adherence to the PLOSNTD Journal Quality Standards on presentation of Tables and Figures.

- We made sure that the tables and figures are in line with the journal guidelines.

  2. In vivo Outcome
  - Line 431-432 Suggested Title: A pharmacokinetic-pharmacodynamic (PK/PD) mismatch
- Thanks for this suggestion, which we implemented (now lane 471).

- Lines 433-535 - Delete. Move to Methodology or re-phrase

- As suggested, these lines were deleted.

3. Comparison of In Vitro Efficacy and In Vivo Outcome
=> A comparison of the results achieved in vitro and in vivo is presented in the Discussion section (lane 485 onwards). Comparing in vitro and in vivo data already in the results section makes no sense.

Review the legend information for each figure (e.g Fig. 1) and remove information that is not necessary, for clarity.

E.g.
Legend: Lines 830-843
Fig 1. Niclosamide and NEN damage E. multilocularis metacestode vesicles and reduce the viability of GL cells in vitro.

Suggested Title: Fig. 1. Effect of niclosamide and NEN on E. multilocularis metacestode vesicles in vitro

- We adapted almost as suggested, but kept the GL in the title, as this is presented here too.

This legend is too long, needs to be reviewed. Most of the information on methodology is not necessary.

- We shortened, but disagree, as we consider that figure legends should remain sufficiently self-explanatory to allow interpretation without referring back to the Methods section.

A, PGI release assay was employed after 5 days of metacestode vesicle treatment to assess the efficacy of
niclosamide (dark grey) and NEN (grey) - both at concentrations ranging from 10’000 to 13.7 nM. DMSO solvent controls are subtracted, and all data is presented relative to the PGI values obtained in the presence of 0.1% Tx-100. Four independent assays were conducted each with technical triplicates. Mean values and SDs from all experiments performed are shown. B, representative light microscopic images of metacestode vesicles treated with 370 nM niclosamide, NEN, and ethanolamine, DMSO (0.1%) and Tx-100 (0.1%). Scale bar = 2 mm. C, GL cell viability as assessed by CellTiter Glo assay upon treatment of GL cells for 5 days with a concentration range (10’000 to 4.9 nM) of niclosamide (dark grey), NEN (grey) or ethanolamine (light grey). Data is shown relative to values obtained upon treatment with 0.1% DMSO (100% viability), and mean values and SDs of four independent experiments are shown, all conducted in technical quadruplicates. D, Representative photomicrographs of drug-treated and control-treated GL cells are shown. Scale bar = 100 µm.

**Conclusions**


Reviewer #1: The conclusions follow from the presented data/ results and are likewise correct and comprehensible.

Reviewer #2: conclusions are supported by the data collected. and the limitations of the study are clearly described

Reviewer #3: Yes, conclusions are supported by the data presented.

- Thank you for the overall positive feedback

As observed and documented in the Discussion: Line 500-503 - "..treatments with both NEN and ESI-09, and several other compounds active against metacestodes, led to the accumulation of extracellular-like vesicles in the matrix of the LL (Zumstein et al., 2025). This response may represent a broader stress reaction of the parasite and warrants further investigation"

Metacestodes actively modulate the host's immune system to survive by triggering an antibody response. This is done by suppressing cell-mediated immunity, absorbing host antibodies on their surface to disguise themselves, and secreting antigens that "trap" the body's antibodies, making it difficult for the immune system to destroy the cysts.
Could these cysts be related to the accumulation of extracellular-like vesicles observed and reported in this study?

- Thank you for this valuable comment. The nature of these vesicles remains unclear, and the current data do not allow us to draw conclusions regarding their origin or function. As this question was outside the scope of the present study, it was not investigated further. We agree that this is an interesting aspect and we will consider it in future work.

Re-formulating of NEN using nanoparticle-based delivery has been suggested as a solution to the following identified challenges that include poor bioavailability, rapid systemic clearance by the liver, and limited tissue penetration. An investigation into the role of cysts will also be important in efforts to re-purpose NEN.

- Thank you for this comment. We absolutely agree and are currently trying to get funding to follow up on exactly this point.

In addition, pharmacokinetic evaluation using the mouse model has its own limitations, which include low total blood volume, making it difficult to collect the multiple time points needed to create a complete plasma concentration-time curve. This may require search for an alternative animal model in order to generate adequate data required for decision making in efficacy evaluation and dose formulation.

- Thank you for this valuable suggestion. We recognize the potential relevance of this approach and will consider its implementation in future studies. However, given the increasingly stringent regulations governing animal experimentation, we currently do not have a suitable alternative to the mouse model for collecting the blood samples necessary for these investigations.

**Editorial and Data Presentation Modifications?**

minor modifications

Reviewer #1: (No Response)

Reviewer #2: An effective drug for treating alveolar echinococcosis is still needed.This study systematically evaluated the in vitro and in vivo efficacy of niclosamide ethanolamine (NEN) against alveolar echinococcosis (AE). The authors found that NEN exhibits potent activity against Echinococcus multilocularis metacestode vesicles and primary cells in vitro (IC50 < 0.3 μM), acting as a mitochondrial uncoupler. However, in a mouse model of intraperitoneal infection, oral NEN treatment for 9 weeks did not significantly reduce parasite burden, despite serum drug concentrations exceeding the in vitro IC50 values. Albendazole (ABZ) was effective, but combination with NEN showed no synergy.
Although the experimental design is rigorous and the data are of good quality, and the “in vitro–in vivo disconnect” is an important cautionary observation in antiparasitic drug development, which is may be a drug candidate.

Some aspects should be concerned:
1. The main conclusion of the study is that “NEN is ineffective in vivo”. The authors should address at one of “whys” which are likely as explanations proposed: poor drug penetration? rapid metabolism? with the infection model? If the drug concentrations can be measured in parasite tissue in vivo(I think it can be measured), it may be a novel explanation to the fail, making it impossible to distinguish between “drug does not reach the target” versus “drug reaches the target but is inactive.”

In addition, no metabolic stability assays (e.g., liver microsomes or in vivo metabolite profiling) were performed to determine whether NEN is rapidly inactivated in the body.
And more, no attempt was made to increase the dose or change the route of administration (e.g., intraperitoneal injection) to rule out absorption or distribution issues.

- Thank you for these valuable thoughts on the “why” that we expanded more in the discussion lines 525-558. We cannot conclude that NEN is ineffective in vivo. But we can conclude that under the conditions used in this experiment, NEN did not exert meaningful activity.
- Performance of metabolic stability assays was not possible within the frame of the current study. However, it has been shown earlier that rapid metabolization of the compound occurs, and to increase exposure, the drug was applied twice daily.
- Increasing the dosage can be done in follow-up studies when additionally alternative formulations of the compound will be applied. One has to keep in mind that this can also cause toxicity issues. ABZ, used as a positive control, however, did exert strong activity under the conditions used in this study.
- We thank you for the input with regard to routes of drug delivery. Intraperitoneal injection of a compound on a daily basis over a period of several weeks is not a viable method, would circumvent critical parameters such as absorption and metabolization, and would also not be applied in human medicine. From an animal welfare point of view, and also keeping in mind that the target population for a new treatment for echinococcosis are human patients, a non-invasive and thus oral formulation would be a preferred option to ensure compliance.

2. The animal model choice has fundamental limitations that undermine the generalizability of the negative result
The study used an intraperitoneal (i.p.) injection model rather than a more natural oral or intrahepatic infection model. In the i.p. model, parasites grow as free vesicle clusters in the peritoneal cavity, lacking the blood supply and stromal environment of normal liver tissue. For a drug like NEN that is known to have low oral bioavailability, high protein binding, and poor tissue penetration (acknowledged by the authors), the i.p. model is very likely to underestimate its potential efficacy. Although the authors mention in the Discussion that an intrahepatic model would be better, they did not use it. The authors should discuss with data using intrahepatic model(s).

- We do not generalize the “negative” results but clearly state that under the conditions used in this study, albendazole is highly active while the NEN formulation we have applied is not. Infection of mice with E. multilocularis eggs is not a trivial issue and is associated with significant biosafety risks. Eggs have to be recovered from wild foxes at high numbers. These eggs are not “standardized”, and viability and infectivity can vary between batches obtained from different animals. We are currently working on refining the egg-infection model, and plan definitely to test NEN in the egg-model as well!
- We have also extended the title of the manuscript to be clearer about the limitation of the infection model.

The intraperitoneal model used in this study represents an advanced stage of disease, with disseminated parasite tissue in the peritoneal cavity (as written in lines 545-550). We disagree with the statement that blood supply is lacking, as even intraperitoneal cysts commonly recruit blood vessels (as documented in Stadelmann et al, 2010).

- ABZ clearly displays the expected efficacy in this model, thus the model per se is working (emphasized now in line 557).

3. The scientific value of the negative result does not meet the bar for publication in PLoS NTDs

PLoS NTDs, as a highimpact journal in the tropical diseases field, prefers studies with positive findings, mechanistic novelty, or clear translational potential. Although the negative result is honest, similar “active in vitro but ineffective in vivo” observations have been reported multiple times for AE drug candidates (e.g., nitazoxanide, mefloquine, buparvaquone). This finding is neither novel nor rare. Moreover, the study does not offer a generalizable solution or a methodological breakthrough (e.g., an in vitro marker that predicts in vivo failure, or a novel formulation strategy).

- We thank the reviewer for this comment. We agree that the absence of efficacy in the in vivo model limits conclusions regarding the therapeutic potential of the compound, as we also outline in the discussion.
- However, we respectfully disagree that the negative in vivo result alone constitutes a reason for non-publication. PLOS Neglected Tropical Diseases evaluates manuscripts based on scientific rigor and whether the conclusions are justified by the evidence presented. Furthermore, the journal's guidelines explicitly state that all relevant positive and negative findings should be reported.
- Our study provides evidence of reproducible in vitro activity and includes mechanistic investigations supporting the proposed mode of action. The negative in vivo findings are reported transparently and are an important component of the overall study. We have carefully restricted our conclusions to reflect the full dataset and do not claim in vivo efficacy or therapeutic utility, but clearly state that NEN does not display in vivo efficacy under the conditions used in this study. We emphasized this more now in lines 37 and 535.
- Moreover, the findings presented here provide a valuable foundation for the future optimization of niclosamide- and NEN-based therapeutic approaches. In particular, the observed in vitro activity together with the mechanistic insights obtained in this study may inform the development of improved formulations and delivery strategies aimed at overcoming the limitations observed in vivo (lines 564 ff). As such, the negative in vivo findings do not diminish the value of the current work but rather help identify critical challenges that should be addressed in subsequent translational studies.
- We therefore believe that the manuscript provides scientifically valuable information to the field, including both its positive mechanistic findings and its negative in vivo results.

4. Flaws in experimental details and statistical analysis
Statistical concerns: In Figure 5, the mean parasite weight in the NEN group (6.31 g) was even higher than in the vehicle control group (4.87 g), albeit not statistically significant. The authors do not discuss whether this suggests a progrowth trend or baseline infection imbalance. No pretreatment parasite burden data are provided, so the success of randomization cannot be assessed.

- We agree that there is a slight trend of a difference that is, however, not significant. We refrain from any speculations at this point and included a respective comment in lines 535-537.

Disconnect between TEM and functional data: No obvious mitochondrial ultrastructural damage was observed by TEM, yet Seahorse and TMRE assays indicated an uncoupling effect. The authors do not explain this discrepancy (it could be due to low concentration or short exposure time, but TEM was performed at 5 days, when structural changes would be expected if the effect were sustained).

- This disconnect is not all surprising and has been found widespread in the literature. The membrane potential is an **electrochemical property**, not a structural one. Electron microscopy visualizes membranes, cristae, and matrix organization, but a mitochondrion can lose much of its potential while retaining an apparently normal ultrastructure. Changes depend on the cell type, with some cell types tolerating mitochondrial depolarization better than others, and the availability of glycolytic ATP and other compensatory mechanisms influences can impaxct on structural damage. in a previous study (Zumstein et al., 2025), where we assessed another uncoupler, we observed exactly the same. Later timepoints were not included, as the entire GL structure is disintegrated at later timepoints. We commented on this further in lines 511-516.

Reviewer #3: A lot of data has been generated from both In Vitro and In Vivo experiments. There is no suggested change in the data as presented, however, re-organization is necessary e.g. methodology repeated in some areas of the results, some statements will require re-phrasing. The methodology section to be presented under three headings: 1) Chemicals and Reagents, 2) In Vitro Efficacy and, 3) In Vivo Outcome, to align with the manuscript Title.
In the Results, there should be an additional heading, no. 4, where a Comparison is made between in Vitro Efficacy and the In Vivo Outcome. These changes will not only align the Methodology, Results, Discussion and Conclusion with the Title for clarity, but also the overall objective of re-purposing NEN.

- Thank you for this valuable suggestion. We were previously unaware that numbered section headings were acceptable for this journal. As we agree that they improve the structure and readability of the manuscript, we have now implemented them throughout the text.
- We will not add an additional chapter on in vitro/in vivo comparison, as the data is presented well in the chapters before and these are no additional results. A comparison on in vitro/in vivo is provided in the discussion.

  Raw, un-analyzed data (in Excel) covering the various treatment groups has been presented. Tables should strictly adhere to the Quality Standards of PLOSNTD Journal.

  Review legends of figures and Tables presented in the Results Section to align to Journal Standards.

**Summary and General Comments**

Reviewer #1: (No Response)

Reviewer #2: (No Response)

Reviewer #3: Overall Comment:
The problem is well defined with a clear objective of re-purposing NEN, for the treatment of Alveolar Echinococcosis, a Neglected Tropical Disease, that has no effective treatments in humans. NEN has been investigated for the inhibition of growth of tumors by slowing down cancer cell metabolism. In the current study, data has been generated using the methodologies described, however, there is need to reorganize it in terms of presentation and alignment to the main objective and the manuscript title.
