# Supplementary material for: Repurposing niclosamide ethanolamine for alveolar echinococcosis reveals a disconnect between in vitro efficacy and in vivo outcome in the intraperitoneal infection model
Source: PLoS Negl Trop Dis. 2026 Aug 31;20(8):e0014016. doi: 10.1371/journal.pntd.0014016 (PMC13552948; doi:10.1371/journal.pntd.0014016)
Supplement: Peer review history

# Peer review: Decision Letter 0

**DOI:** 10.1371/journal.pntd.0014016.r001

**Authors:** Krystyna Cwiklinski, Ramon Eichenberger

10 Jun 2026

PNTD-D-26-00251

Repurposing Niclosamide Ethanolamine for Alveolar Echinococcosis Reveals a Disconnect Between In Vitro Efficacy and In Vivo Outcome

PLOS Neglected Tropical Diseases

Dear Dr. Lundström-Stadelmann,

Thank you for submitting your manuscript to PLOS Neglected Tropical Diseases. After careful consideration, we feel that it has merit but does not fully meet PLOS Neglected Tropical Diseases's publication criteria as it currently stands. Therefore, we invite you to submit a revised version of the manuscript that addresses the points raised during the review process.

Please submit your revised manuscript within by Aug 09 2026 11:59PM. If you will need more time than this to complete your revisions, please reply to this message or contact the journal office at plosntds@plos.org. When you're ready to submit your revision, log on to https://www.editorialmanager.com/pntd/ and select the 'Submissions Needing Revision' folder to locate your manuscript file.

Please include the following items when submitting your revised manuscript:

* A letter that responds to each point raised by the editor and reviewer(s). You should upload this letter as a separate file labeled 'Response to Reviewers'. This file does not need to include responses to any formatting updates and technical items listed in the 'Journal Requirements' section below.

* A marked-up copy of your manuscript that highlights changes made to the original version. You should upload this as a separate file labeled 'Revised Manuscript with Track Changes'.

* An unmarked version of your revised paper without tracked changes. You should upload this as a separate file labeled 'Manuscript'.

If you would like to make changes to your financial disclosure, competing interests statement, or data availability statement, please make these updates within the submission form at the time of resubmission. Guidelines for resubmitting your figure files are available below the reviewer comments at the end of this letter.

As the corresponding author, your ORCID iD is verified in the submission system and will appear in the published article. PLOS supports the use of ORCID, and we encourage all coauthors to register for an ORCID iD and use it as well. Please encourage your coauthors to verify their ORCID iD within the submission system before final acceptance, as unverified ORCID iDs will not appear in the published article. Only the individual author can complete the verification step; PLOS staff cannot verify ORCID iDs on behalf of authors.

We look forward to receiving your revised manuscript.

Kind regards,

Ramon Marc Eichenberger

Academic Editor

PLOS Neglected Tropical Diseases

Krystyna Cwiklinski

Section Editor

PLOS Neglected Tropical Diseases

Shaden Kamhawi

co-Editor-in-Chief

PLOS Neglected Tropical Diseases

orcid.org/0000-0003-4304-636XX

Paul Brindley

co-Editor-in-Chief

PLOS Neglected Tropical Diseases

orcid.org/0000-0003-1765-0002

**Additional Editor Comments (if provided):**

**Journal Requirements:**

If the reviewer comments include a recommendation to cite specific previously published works, please review and evaluate these publications to determine whether they are relevant and should be cited. There is no requirement to cite these works unless the editor has indicated otherwise.

1) Please ensure that the CRediT author contributions listed for every co-author are completed accurately and in full.

At this stage, the following Authors/Authors require contributions: Matías Preza, Nicole Dietrich, Pascal Zumstein, Judith Steinmann, Lea Hiller, Trix Zumkehr, Tobias Kämpfer, Marylène Chollet-Krugler, Laura Vetter, Andrew Hemphill, Sarah Dion, and Britta Lundström-Stadelmann. Please ensure that the full contributions of each author are acknowledged in the "Add/Edit/Remove Authors" section of our submission form.

The list of CRediT author contributions may be found here: https://journals.plos.org/plosntds/s/authorship#loc-author-contributions

2) We do not publish any copyright or trademark symbols that usually accompany proprietary names, eg ©,  ®, or TM  (e.g. next to drug or reagent names). Therefore please remove all instances of trademark/copyright symbols throughout the text, including:

- ® on pages: 12, and 13

- TM on pages: 10, 13, and 14.

3) Please amend your detailed Financial Disclosure statement. This is published with the article. It must therefore be completed in full sentences and contain the exact wording you wish to be published.

i) State what role the funders took in the study. If the funders had no role in your study, please state: "The funders had no role in study design, data collection and analysis, decision to publish, or preparation of the manuscript."

ii) If any authors received a salary from any of your funders, please state which authors and which funders..

If you did not receive any funding for this study, please simply state: u201cThe authors received no specific funding for this work.u201d

4) We have amended your Competing Interest statement to comply with journal style. We kindly ask that you double check the statement and let us know if anything is incorrect.

5) Your current Financial Disclosure states, “This study was supported by grants to BLS from the Uniscientia Foundation and the Gottfried and Julia Bangerter-Rhyner Foundation, as well as the Swiss National Science Foundation (SNSF) grant number 320030-227438 and 320030-236056. The funders had no role in study design, data collection, analysis and interpretation, decision to publish, or preparation of the manuscript.” However, in your funding information on the submission form, "order of grants" mismatches. Please indicate by return email the full and correct funding information for your study and confirm the order in which funding contributions should appear. Please be sure to indicate whether the funders played any role in the study design, data collection and analysis, decision to publish, or preparation of the manuscript.

**Reviewers' Comments:**

Reviewer's Responses to Questions

**Key Review Criteria Required for Acceptance?**

As you describe the new analyses required for acceptance, please consider the following:

**Methods**

-Are the objectives of the study clearly articulated with a clear testable hypothesis stated?

-Is the study design appropriate to address the stated objectives?

-Is the population clearly described and appropriate for the hypothesis being tested?

-Is the sample size sufficient to ensure adequate power to address the hypothesis being tested?

-Were correct statistical analysis used to support conclusions?

-Are there concerns about ethical or regulatory requirements being met?

Reviewer #1: The present study investigating niclosamide and NEN, assessing the effects of these compounds on Echinococcus multilocularis both in vitro and in vivo in a mouse model, can be considered relevant and is of very high quality in terms of methodology and study design.

Reviewer #2: The experimental design is rigorous with clearly articulated. and the data are of good quality.

Reviewer #3: The objective is well articulated; that of repurposing NEN from the field of oncology to the treatment of echinococcosis. This is based on the fact that previous studies have demonstrated high liver concentrations of 1.5 µM within 4 h in mice treated with 40 mg/kg. This is in contrast to niclosamide, an FDA-approved anthelmintic that has shown poor gastrointestinal absorption and and bioavailability preventing effective penetration and elimination of tissue-dwelling parasites.

Ethical approval was obtained for the conduct of the study. Requirements were met.

Study design: Evaluation of NEN and other available drugs to determine the In Vitro Efficacy and In Vivo Outcomes was well planned.

The authors to consider presentation of the methodology section under two headings for clarity: In Vitro Efficacy and In Vivo Outcome. This will avoid repetitions and enhance information flow and readership. In some areas, especially grouping of experimental animals, I suggest that tables be used. These will also assist in presentation of results/findings for clarity.

All tables: must strictly adhere to PLONTD Journal standards. Each Table should self explanatory, with clear title. Any abbreviations used should be explained in the table legend.

Methodology:

1. Chemicals and Reagents

2. In vitro Efficacy

Parasite source and maintenance (Lines 146-148)

Line 146: Provide a short description of are the H95 parasites? Indicate source.

In vitro culture of metacestode vesicles (lines 149-155)

Assessment of NEN-induced metacestode vesicle damage (lines 157-158; delete "..by phosphoglucose isomerase

assay"). This is well-captured in the text.

E. multilocularis GL cell isolation (line 176)

- Replace GL with germinal layer in the title)

Methods on in vitro Efficacy to be included in this section are well detailed.

2). At Line 284 insert: In vivo Outcome

- Experimental animals; to include ethical clearance, description, source, and maintenance

Re-organize this section (refer to In Vitro Efficacy section).

**********

**Results**

-Does the analysis presented match the analysis plan?

-Are the results clearly and completely presented?

-Are the figures (Tables, Images) of sufficient quality for clarity?

Reviewer #1: The results are presented in a structured and clear manner, and the conclusions drawn are appropriate and well supported by the data. The data, figures, and tables are adequate and correctly labeled.

Reviewer #2: The results are clearly presented, especially, the “in vitro–in vivo disconnect” is an important cautionary observation in antiparasitic drug development.

Reviewer #3: Suggestion: that the presentation of Results follows the re-organized Methodology section.

As presented, the results are detailed. Re-organization of the text will avoid repetition of methodology. This will also assist in aligning the Discussion and Conclusion not only to the main Objective of the study but also the Manuscript Title: "Repurposing Niclosamide Ethanolamine for Alveolar Echinococcosis Reveals a Disconnect Between In Vitro Efficacy and In Vivo Outcome".

Results:

1. In vitro Efficacy

- Line 365 information already in methodology. Either delete or re-phrase to read " Following assessment of the efficacy of ...using PGI assay,...."

Line 397: Ensure Table 1 is in strict adherence to the PLOSNTD Journal Quality Standards on presentation of Tables and Figures.

2. In vivo Outcome

- Line 431-432 Suggested Title: A pharmacokinetic-pharmacodynamic (PK/PD) mismatch

- Lines 433-535 - Delete. Move to Methodology or re-phrase

3. Comparison of In Vitro Efficacy and In Vivo Outcome

Review the legend information for each figure (e.g Fig. 1) and remove information that is not necessary, for clarity.

E.g.

Legend: Lines 830-843

Fig 1. Niclosamide and NEN damage E. multilocularis metacestode vesicles and reduce the viability of GL cells in vitro.

Suggested Title: Fig. 1. Effect of niclosamide and NEN on E. multilocularis metacestode vesicles in vitro

This legend is too long, needs to be reviewed. Most of the information on methodology is not necessary.

A, PGI release assay was employed after 5 days of metacestode vesicle treatment to assess the efficacy of

niclosamide (dark grey) and NEN (grey) - both at concentrations ranging from 10’000 to 13.7 nM. DMSO solvent controls are subtracted, and all data is presented relative to the PGI values obtained in the presence of 0.1% Tx-100. Four independent assays were conducted each with technical triplicates. Mean values and SDs from all experiments performed are shown. B, representative light microscopic images of metacestode vesicles treated with 370 nM niclosamide, NEN, and ethanolamine, DMSO (0.1%) and Tx-100 (0.1%). Scale bar = 2 mm. C, GL cell viability as assessed by CellTiter Glo assay upon treatment of GL cells for 5 days with a concentration range (10’000 to 4.9 nM) of niclosamide (dark grey), NEN (grey) or ethanolamine (light grey). Data is shown relative to values obtained upon treatment with 0.1% DMSO (100% viability), and mean values and SDs of four independent experiments are shown, all conducted in technical quadruplicates. D, Representative photomicrographs of drug-treated and control-treated GL cells are shown. Scale bar = 100 µm.

**********

**Conclusions**

-Are the conclusions supported by the data presented?

-Are the limitations of analysis clearly described?

-Do the authors discuss how these data can be helpful to advance our understanding of the topic under study?

-Is public health relevance addressed?

Reviewer #1: The conclusions follow from the presented data/ results and are likewise correct and comprehensible.

Reviewer #2: conclusions are supported by the data collected. and the limitations of the study are clearly described

Reviewer #3: Yes, conclusions are supported by the data presented.

As observed and documented in the Discussion: Line 500-503 - "..treatments with both NEN and ESI-09, and several other compounds active against metacestodes, led to the accumulation of extracellular-like vesicles in the matrix of the LL (Zumstein et al., 2025). This response may represent a broader stress reaction of the parasite and warrants further investigation"

Metacestodes actively modulate the host's immune system to survive by triggering an antibody response. This is done by suppressing cell-mediated immunity, absorbing host antibodies on their surface to disguise themselves, and secreting antigens that "trap" the body's antibodies, making it difficult for the immune system to destroy the cysts.

Could these cysts be related to the accumulation of extracellular-like vesicles observed and reported in this study?

Re-formulating of NEN using nanoparticle-based delivery has been suggested as a solution to the following identified challenges that include poor bioavailability, rapid systemic clearance by the liver, and limited tissue penetration. An investigation into the role of cysts will also be important in efforts to re-purpose NEN.

In addition, pharmacokinetic evaluation using the mouse model has its own limitations, which include low total blood volume, making it difficult to collect the multiple time points needed to create a complete plasma concentration-time curve. This may require search for an alternative animal model in order to generate adequate data required for decision making in efficacy evaluation and dose formulation.

**********

**Editorial and Data Presentation Modifications?**

Use this section for editorial suggestions as well as relatively minor modifications of existing data that would enhance clarity. If the only modifications needed are minor and/or editorial, you may wish to recommend “Minor Revision” or “Accept”.

Reviewer #1: (No Response)

Reviewer #2: An effective drug for treating alveolar echinococcosis is still needed.This study systematically evaluated the in vitro and in vivo efficacy of niclosamide ethanolamine (NEN) against alveolar echinococcosis (AE). The authors found that NEN exhibits potent activity against Echinococcus multilocularis metacestode vesicles and primary cells in vitro (IC50 < 0.3 μM), acting as a mitochondrial uncoupler. However, in a mouse model of intraperitoneal infection, oral NEN treatment for 9 weeks did not significantly reduce parasite burden, despite serum drug concentrations exceeding the in vitro IC50 values. Albendazole (ABZ) was effective, but combination with NEN showed no synergy.

Although the experimental design is rigorous and the data are of good quality, and the “in vitro–in vivo disconnect” is an important cautionary observation in antiparasitic drug development, which is may be a drug candidate.

Some aspects should be concerned:

1. The main conclusion of the study is that “NEN is ineffective in vivo”. The authors should address at one of “whys” which are likely as explanations proposed: poor drug penetration? rapid metabolism? with the infection model? If the drug concentrations can be measured in parasite tissue in vivo(I think it can be measured), it may be a novel explanation to the fail, making it impossible to distinguish between “drug does not reach the target” versus “drug reaches the target but is inactive.”

In addition, no metabolic stability assays (e.g., liver microsomes or in vivo metabolite profiling) were performed to determine whether NEN is rapidly inactivated in the body.

And more, no attempt was made to increase the dose or change the route of administration (e.g., intraperitoneal injection) to rule out absorption or distribution issues.

2. The animal model choice has fundamental limitations that undermine the generalizability of the negative result

The study used an intraperitoneal (i.p.) injection model rather than a more natural oral or intrahepatic infection model. In the i.p. model, parasites grow as free vesicle clusters in the peritoneal cavity, lacking the blood supply and stromal environment of normal liver tissue. For a drug like NEN that is known to have low oral bioavailability, high protein binding, and poor tissue penetration (acknowledged by the authors), the i.p. model is very likely to underestimate its potential efficacy. Although the authors mention in the Discussion that an intrahepatic model would be better, they did not use it. The authors should discuss with data using intrahepatic model(s).

3. The scientific value of the negative result does not meet the bar for publication in PLoS NTDs

PLoS NTDs, as a highimpact journal in the tropical diseases field, prefers studies with positive findings, mechanistic novelty, or clear translational potential. Although the negative result is honest, similar “active in vitro but ineffective in vivo” observations have been reported multiple times for AE drug candidates (e.g., nitazoxanide, mefloquine, buparvaquone). This finding is neither novel nor rare. Moreover, the study does not offer a generalizable solution or a methodological breakthrough (e.g., an in vitro marker that predicts in vivo failure, or a novel formulation strategy).

4. Flaws in experimental details and statistical analysis

Statistical concerns: In Figure 5, the mean parasite weight in the NEN group (6.31 g) was even higher than in the vehicle control group (4.87 g), albeit not statistically significant. The authors do not discuss whether this suggests a progrowth trend or baseline infection imbalance. No pretreatment parasite burden data are provided, so the success of randomization cannot be assessed.

Disconnect between TEM and functional data: No obvious mitochondrial ultrastructural damage was observed by TEM, yet Seahorse and TMRE assays indicated an uncoupling effect. The authors do not explain this discrepancy (it could be due to low concentration or short exposure time, but TEM was performed at 5 days, when structural changes would be expected if the effect were sustained).

Reviewer #3: A lot of data has been generated from both In Vitro and In Vivo experiments. There is no suggested change in the data as presented, however, re-organization is necessary e.g. methodology repeated in some areas of the results, some statements will require re-phrasing. The methodology section to be presented under three headings: 1) Chemicals and Reagents, 2) In Vitro Efficacy and, 3) In Vivo Outcome, to align with the manuscript Title.

In the Results, there should be an additional heading, no. 4, where a Comparison is made between in Vitro Efficacy and the In Vivo Outcome. These changes will not only align the Methodology, Results, Discussion and Conclusion with the Title for clarity, but also the overall objective of re-purposing NEN.

Raw, un-analyzed data (in Excel) covering the various treatment groups has been presented. Tables should strictly adhere to the Quality Standards of PLOSNTD Journal.

Review legends of figures and Tables presented in the Results Section to align to Journal Standards.

**********

**Summary and General Comments**

Use this section to provide overall comments, discuss strengths/weaknesses of the study, novelty, significance, general execution and scholarship. You may also include additional comments for the author, including concerns about dual publication, research ethics, or publication ethics. If requesting major revision, please articulate the new experiments that are needed.

Reviewer #1: (No Response)

Reviewer #2: (No Response)

Reviewer #3: Overall Comment:

The problem is well defined with a clear objective of re-purposing NEN, for the treatment of Alveolar Echinococcosis, a Neglected Tropical Disease, that has no effective treatments in humans. NEN has been investigated for the inhibition of growth of tumors by slowing down cancer cell metabolism. In the current study, data has been generated using the methodologies described, however, there is need to reorganize it in terms of presentation and alignment to the main objective and the manuscript title.

**********

PLOS authors have the option to publish the peer review history of their article (what does this mean?). If published, this will include your full peer review and any attached files.

If you choose “no”, your identity will remain anonymous but your review may still be made public.

**Do you want your identity to be public for this peer review?**  For information about this choice, including consent withdrawal, please see our Privacy Policy.

Reviewer #1: **Yes:** Beate Grüner

Reviewer #2: No

Reviewer #3: **Yes:** Prof. Grace Adira Murilla

[NOTE: If reviewer comments were submitted as an attachment file, they will be attached to this email and accessible via the submission site. Please log into your account, locate the manuscript record, and check for the action link "View Attachments". If this link does not appear, there are no attachment files.]

**Figure resubmission:**

**Reproducibility:**

To enhance the reproducibility of your results, we recommend that authors of applicable studies deposit laboratory protocols in protocols.io, where a protocol can be assigned its own identifier (DOI) such that it can be cited independently in the future. Additionally, PLOS ONE offers an option to publish peer-reviewed clinical study protocols. Read more information on sharing protocols at https://plos.org/protocols?utm_medium=editorial-email&utm_source=authorletters&utm_campaign=protocols

Attachment Submitted filename: Comments for Manuscript No. PNTD-D-26-00251.docx

# Author comment: Author response to Decision Letter 1

**DOI:** 10.1371/journal.pntd.0014016.r002

22 Jun 2026

Attachment Submitted filename: Rebuttle letter.docx

# Peer review: Decision Letter 1

**DOI:** 10.1371/journal.pntd.0014016.r003

**Authors:** Krystyna Cwiklinski, Ramon Eichenberger

19 Aug 2026

Dear Prof. Dr. Lundström-Stadelmann,

We are pleased to inform you that your manuscript 'Repurposing Niclosamide Ethanolamine for Alveolar Echinococcosis Reveals a Disconnect Between In Vitro Efficacy and In Vivo Outcome in the Intraperitoneal Infection Model' has been provisionally accepted for publication in PLOS Neglected Tropical Diseases.

Before your manuscript can be formally accepted you will need to complete some formatting changes, which you will receive in a follow up email. A member of our team will be in touch with a set of requests.

Please note that your manuscript will not be scheduled for publication until you have made the required changes, so a swift response is appreciated.

IMPORTANT: The editorial review process is now complete. PLOS will only permit corrections to spelling, formatting or significant scientific errors from this point onwards. Requests for major changes, or any which affect the scientific understanding of your work, will cause delays to the publication date of your manuscript.

Should you, your institution's press office or the journal office choose to press release your paper, you will automatically be opted out of early publication. We ask that you notify us now if you or your institution is planning to press release the article. All press must be co-ordinated with PLOS.

Thank you again for supporting Open Access publishing; we are looking forward to publishing your work in PLOS Neglected Tropical Diseases.

Best regards,

Ramon Marc Eichenberger

Academic Editor

PLOS Neglected Tropical Diseases

Krystyna Cwiklinski

Section Editor

PLOS Neglected Tropical Diseases

Shaden Kamhawi

co-Editor-in-Chief

PLOS Neglected Tropical Diseases

orcid.org/0000-0003-4304-636XX

Paul Brindley

co-Editor-in-Chief

PLOS Neglected Tropical Diseases

orcid.org/0000-0003-1765-0002

# Editor report: Acceptance letter

**DOI:** 10.1371/journal.pntd.0014016.r004

**Authors:** Krystyna Cwiklinski, Ramon Eichenberger

Dear Prof. Dr. Lundström-Stadelmann,

We are delighted to inform you that your manuscript, "Repurposing Niclosamide Ethanolamine for Alveolar Echinococcosis Reveals a Disconnect Between In Vitro Efficacy and In Vivo Outcome in the Intraperitoneal Infection Model," has been formally accepted for publication in PLOS Neglected Tropical Diseases.

We have now passed your article onto the PLOS Production Department who will complete the rest of the publication process. All authors will receive a confirmation email upon publication.

The corresponding author will soon be receiving a typeset proof for review, to ensure errors have not been introduced during production. Please review the PDF proof of your manuscript carefully, as this is the last chance to correct any scientific or type-setting errors. Please note that major changes, or those which affect the scientific understanding of the work, will likely cause delays to the publication date of your manuscript. Note: Proofs for Front Matter articles (Editorial, Viewpoint, Symposium, Review, etc...) are generated on a different schedule and may not be made available as quickly.

Soon after your final files are uploaded, the early version of your manuscript will be published online unless you opted out of this process. The date of the early version will be your article's publication date. The final article will be published to the same URL, and all versions of the paper will be accessible to readers.

For Research Articles, you will receive an invoice from PLOS for your publication fee after your manuscript has reached the completed accept phase. If you receive an email requesting payment before acceptance or for any other service, this may be a phishing scheme. Learn how to identify phishing emails and protect your accounts at https://explore.plos.org/phishing.

Thank you again for supporting open-access publishing; we are looking forward to publishing your work in PLOS Neglected Tropical Diseases.

Best regards,

Shaden Kamhawi

co-Editor-in-Chief

PLOS Neglected Tropical Diseases

Paul Brindley

co-Editor-in-Chief

PLOS Neglected Tropical Diseases
